# High-resolution three-dimensional quantitative map of the macromolecular proton fraction distribution in the normal rat brain

**DOI:** 10.1016/j.dib.2016.11.066

**Published:** 2016-12-05

**Authors:** Anna V. Naumova, Andrey E. Akulov, Marina Yu. Khodanovich, Vasily L. Yarnykh

**Affiliations:** a University of Washington, Department of Radiology, 850 Republican St., Seattle, WA, USA; b National Research Tomsk State University, Research Institute of Biology and Biophysics, 36 Lenina Ave, Tomsk, Russia; c Institute of Cytology and Genetics, The Siberian Branch of the Russian Academy of Sciences, Prospekt Lavrentyeva 10, Novosibirsk, Russia

**Keywords:** MPF, macromolecular proton fraction, MRI, magnetic resonance imaging, T, Tesla, 3D, three dimensional, MT, magnetization transfer, PD, proton density, T_1_, longitudinal relaxation time

## Abstract

The presented dataset provides a normative high-resolution three-dimensional (3D) macromolecular proton fraction (MPF) map of the healthy rat brain in vivo and source images used for its reconstruction. The images were acquired using the protocol described elsewhere (Naumova, et al. High-resolution three-dimensional macromolecular proton fraction mapping for quantitative neuroanatomical imaging of the rodent brain in ultra-high magnetic fields. Neuroimage (2016) doi: 10.1016/j.neuroimage.2016.09.036). The map was reconstructed from three source images with different contrast weightings (proton density, T_1_, and magnetization transfer) using the single-point algorithm with a synthetic reference image. Source images were acquired from a living animal on an 11.7 T small animal MRI scanner with isotropic spatial resolution of 170 µm^3^ and total acquisition time about 1.5 h. The 3D dataset can be used for multiple purposes including interactive viewing of rat brain anatomy, measurements of reference MPF values in various brain structures, and development of image processing techniques for the rodent brain segmentation. It also can serve as a gold standard image for implementation and optimization of rodent brain MRI protocols.

**Specifications Table**

**Table:** 

Subject area	*Neuroimaging*
More specific subject area	*Small animal brain MRI*
Type of data	*3D images, figure*
How data was acquired	11.7 T *MRI scanner (BioSpec* 117/16 *USR; Bruker BioSpin, Ettlingen, Germany)*
Data format	*Analyze* 7.5
Experimental factors	*Living healthy animal; no pretreatment.*
Experimental features	*in vivo magnetic resonance imaging of the rat brain*
*Data source location*	*Institute of Cytology and Genetics, The Siberian Branch of the Russian Academy of Sciences, Novosibirsk, Russia.*
Data accessibility	*Data is within this article*

**Value of the data**

• The presented dataset provides a normative high-resolution three-dimensional (3D) macromolecular proton fraction (MPF) map of the healthy rat brain in vivo.
• The 3D dataset can be used for multiple purposes including interactive viewing of the rat brain anatomy, measurements of reference MPF values in various brain structures, and development of image processing techniques for the rodent brain segmentation.
• The 3D dataset of the normal rat brain can serve as a gold standard for implementation and optimization of rodent brain MRI protocols.

## 1 Data

**Fig. 1:**
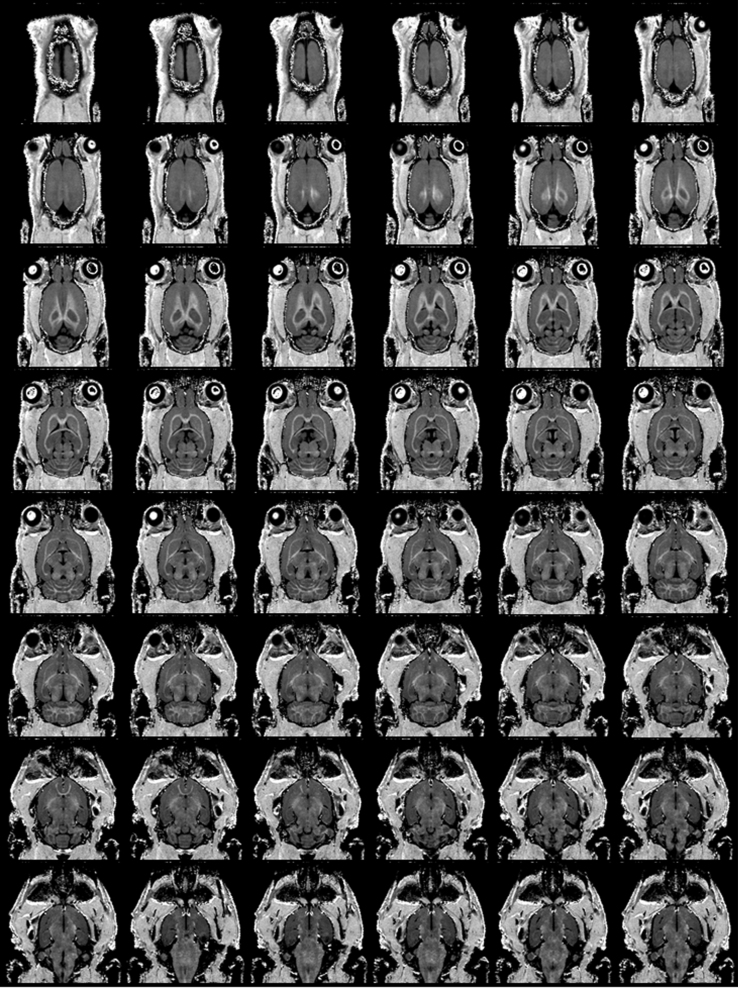
Reformatted coronal sections of a 3D MPF map of the rat brain obtained using the single-point synthetic-reference method with isotropic resolution of 170 µm^3^.

The presented 3D dataset illustrates the application of the fast macromolecular proton fraction (MPF) mapping method [1], [2], [3] for generation of high-contrast high-resolution images of the rodent brain in vivo in ultra-high magnetic fields. The entire dataset contains 3D image files in the Analyze 7.5 format representing three source images with different contrast weightings (proton density (PD): files “PD.img” and “PD.hdr”, T_1_: files “T1.img” and “T1.hdr”, and magnetization transfer (MT): files “MT.img” and “MT.hdr”) of the rat brain in vivo acquired on an 11.7 T small animal MRI scanner and an MPF map (files MPF.img and MPF.hdr) reconstructed from these images. Each 3D image has isotropic spatial resolution of 170×170×170 µm and contains a 200×200×200 matrix with voxel intensities in 16-bit little-endian format. Intensities of PD-, T_1_-, and MT-weighted images are in arbitrary units and MPF map intensities represent percentage MPF values multiplied by 100. Example reformatted coronal sections of the 3D MPF map are presented in Fig. 1.

## 2 Experimental design, materials and methods

Animal procedures were approved by the Bioethical Committee at the Institute of Cytology and Genetics of the Siberian Branch of the Russian Academy of Sciences. An adult male Wistar rat was imaged in vivo on a 11.7 T horizontal-bore animal MRI scanner (BioSpec 117/16 USR; Bruker BioSpin, Ettlingen, Germany) with a four-channel surface phased-array coil. The protocol included three spoiled 3D gradient echo sequences enabling MT, T_1_, and PD contrast weightings. Images were acquired with whole-brain coverage, isotropic voxel size of 170 µm^3^, and the total scan time of about 1.5 h. Details of the imaging protocol can be found elsewhere [1]. The MPF map was reconstructed using custom-written C-language software according to the single-point method [2] with the synthetic reference image for data normalization [3] and two-pool model parameter constraints specifically determined for 11.7 T magnetic field [1].
