# Supplementary material for: High-resolution three-dimensional quantitative map of the macromolecular proton fraction distribution in the normal rat brain
Source: Data Brief. 2016 Dec 5;10:381–4. doi: 10.1016/j.dib.2016.11.066 (PMC5176127; doi:10.1016/j.dib.2016.11.066)
Supplement: Supplementary file 1 — Supplementary material [file mmc1.docx]

The authors have no conflicts of interest regarding this manuscript.
